# The absence of the *Pseudomonas aeruginosa* OprF protein leads to increased biofilm formation through variation in c-di-GMP level

**DOI:** 10.3389/fmicb.2015.00630

**Published:** 2015-06-23

**Authors:** Emeline Bouffartigues, Joana A. Moscoso, Rachel Duchesne, Thibaut Rosay, Laurène Fito-Boncompte, Gwendoline Gicquel, Olivier Maillot, Magalie Bénard, Alexis Bazire, Gerald Brenner-Weiss, Olivier Lesouhaitier, Patrice Lerouge, Alain Dufour, Nicole Orange, Marc G. J. Feuilloley, Joerg Overhage, Alain Filloux, Sylvie Chevalier

**Affiliations:** ^1^EA 4312-Laboratory of Microbiology Signals and Microenvironment, University of Rouen – Normandy UniversityEvreux, France; ^2^MRC Centre for Molecular Bacteriology and Infection, Department of Life Sciences, Imperial College LondonLondon, UK; ^3^Cell Imaging Platform of Normandy (PRIMACEN), Institute for Research and Innovation in Biomedicine, University of RouenMont-Saint-Aignan, France; ^4^EA 3884-Laboratoire de Biotechnologie et Chimie Marines, Institut Universitaire Européen de la Mer, Université de Bretagne-SudLorient, France; ^5^Institute of Functional Interfaces, Karlsruhe Institute of TechnologyKarlsruhe, Germany; ^6^Glyco-MeV Laboratory, University of Rouen, Normandy UniversityMont-Saint-Aignan, France

**Keywords:** OprF, exopolysaccharides, biofilm, c-di-GMP, SigX, ECF

## Abstract

OprF is the major outer membrane porin in bacteria belonging to the *Pseudomonas* genus. In previous studies, we have shown that OprF is required for full virulence expression of the opportunistic pathogen *Pseudomonas aeruginosa*. Here, we describe molecular insights on the nature of this relationship and report that the absence of OprF leads to increased biofilm formation and production of the Pel exopolysaccharide. Accordingly, the level of c-di-GMP, a key second messenger in biofilm control, is elevated in an *oprF* mutant. By decreasing c-di-GMP levels in this mutant, both biofilm formation and *pel* gene expression phenotypes were restored to wild-type levels. We further investigated the impact on two small RNAs, which are associated with the biofilm lifestyle, and found that expression of *rsmZ* but not of *rsmY* was increased in the *oprF* mutant and this occurs in a c-di-GMP-dependent manner. Finally, the extracytoplasmic function (ECF) sigma factors AlgU and SigX displayed higher activity levels in the *oprF* mutant. Two genes of the SigX regulon involved in c-di-GMP metabolism, PA1181 and *adcA* (PA4843), were up-regulated in the *oprF* mutant, partly explaining the increased c-di-GMP level. We hypothesized that the absence of OprF leads to a cell envelope stress that activates SigX and results in a c-di-GMP elevated level due to higher expression of *adcA* and PA1181. The c-di-GMP level can in turn stimulate Pel synthesis via increased *rsmZ* sRNA levels and *pel* mRNA, thus affecting Pel-dependent phenotypes such as cell aggregation and biofilm formation. This work highlights the connection between OprF and c-di-GMP regulatory networks, likely via SigX (ECF), on the regulation of biofilm phenotypes.

## Introduction

*Pseudomonas aeruginosa* is a Gram-negative bacterium found in almost every wet ecological niches including soils, water, and plants (Spiers et al., 2000). It is also an opportunistic human pathogen, causing a variety of infections including chronic lung infections in cystic fibrosis patients (Ramsey and Wozniak, 2005). This requires a particularly well-developed ability to adapt to changes in environmental conditions, in which outer membrane (OM) proteins may play an important function considering their role in communication with the extracellular medium. The major *Pseudomonas* sp. OM protein OprF is homologous to OmpA of Enterobacteriaceae, and is the only general *P. aeruginosa* porin (Tamber and Hancock, 2004). It allows a non-specific diffusion of ionic species (Yoon et al., 2002) and small polar nutrients, including polysaccharides up to 1.5 kDa in size (Nestorovich et al., 2006). OprF anchors the OM to the peptidoglycan layer (Woodruff and Hancock, 1989; Rawling et al., 1998), is involved in host-pathogen interactions, and is required for expression of full virulence (Fito-Boncompte et al., 2011). Binding of human interferon-γ to OprF leads to the expression of the PA-I lectin, a quorum-sensing-dependent virulence determinant, suggesting that OprF acts as a sensor of the host immune system (Wu et al., 2005). OprF has also been proposed to be involved in rhamnolipid production (Bouffartigues et al., 2011), adhesion onto eukaryotic cells (Azghani et al., 2002; Fito-Boncompte et al., 2011), and biofilm development under anaerobic conditions (Hassett et al., 2002; Yoon et al., 2002). OprF expression is controlled by at least two extracytoplasmic function (ECFs) sigma factors, AlgU and SigX (Brinkman et al., 1999; Firoved et al., 2002; Bouffartigues et al., 2012), which are involved in cell envelope homeostasis (Wood et al., 2006; Boechat et al., 2013; Duchesne et al., 2013; Gicquel et al., 2013; Blanka et al., 2014).

Biofilm growth is a bacterial lifestyle of particular importance for *P. aeruginosa* pathogenesis (Ramsey and Wozniak, 2005). These surface-associated microbial communities of cells are embedded into a matrix of extracellular polymeric substances (Donlan and Costerton, 2002; Allesen-Holm et al., 2006; Ma et al., 2009). In *P. aeruginosa*, the matrix contains an array of components such as extracellular DNA (Allesen-Holm et al., 2006; Ma et al., 2009), proteinaceous adhesins, vesicles and exopolysaccharides (EPS; Ryder et al., 2007; Gooderham and Hancock, 2009; Mikkelsen et al., 2011). In several non-mucoid strains, such as the PAO1 strain, Psl and Pel are two key EPS that maintain biofilm structure (Friedman and Kolter, 2004a; Ma et al., 2006; Colvin et al., 2012). Pel is a glucose-rich EPS that is required to form pellicles at the air-liquid interface or mature solid surface-associated biofilms (Friedman and Kolter, 2004a,b; Ghafoor et al., 2011). Psl is a repeating penta-saccharide containing D-mannose, D-glucose and L-rhamnose (Byrd et al., 2009), which is of particular importance for the stability (shear resistance) of non-mucoid biofilms (Bazire et al., 2010). Expression of the *pel* and *psl* gene clusters is highly complex. It is regulated transcriptionally by the second messenger bis-(3′–5′)-cyclic guanosine monophosphate (c-di-GMP; Lee et al., 2007; Starkey et al., 2009; Borlee et al., 2010), and translationally by the Gac/Rsm signaling network (Brencic and Lory, 2009; Irie et al., 2010).

We report here that the absence of OprF in the OM of *P. aeruginosa* results in increased biofilm formation. We show that this is concomitant to (i) the overproduction of Pel but not Psl, (ii) the increase in c-di-GMP levels and (iii) the up-regulation of *rsmZ*, a small non-coding RNA of the Gac/Rsm signaling pathway. The artificial reduction of c-di-GMP in the *oprF* mutant resulted in the restoration of these phenotypes. Two genes of the SigX regulon that are involved in regulating c-di-GMP levels, *adcA* (PA4843; Jones et al., 2014) and PA1181, are up-regulated in the *oprF* mutant, which thus links OprF and c-di-GMP regulatory networks.

## Materials and Methods

### Bacterial Strains and Growth Conditions

The strains are listed in **Table 1**. Strains were grown overnight at 37°C on a rotary shaker (180 rpm) in Luria Bertani (LB) broth. Five hundred μg streptomycin ml^-1^ only or with 300 μg carbenicillin ml^-1^ were added in H636 and H636O precultures, respectively. Under these conditions, the growth of the three strains was closely similar (Fito-Boncompte et al., 2011). When indicated, 40 μg.ml^-1^ of congo red (CR) was added to the LB growth medium. Fifty μg.ml^-1^ of tetracyclin were added to the growth medium for the culture of the H636C complemented strain. The P_cdrA_-gfp, pJN105 and pJN2133 plasmids were introduced into P. aeruginosa strains by electroporation and transformants were selected and grown in LB containing 100 μg gentamicin ml^-1^.

**Table 1:** Bacterial strains and plasmids used in this study.

Strains/plasmids	Relevant characteristic(s)	Source
**Strains**
***Escherichia coli***	
DH5α	Cloning host	Life technology
SM10pir	Host strain for mini-CTX1	de Lorenzo and Timmis (1994)
***Pseudomonas aeruginosa***	
H103	PAO1 derivative	Hancock and Carey (1979)
H636	PAO1 H103 *oprF::Ω*	Woodruff and Hancock (1988)
H636O	pRW5 containing H636 strain	Woodruff and Hancock (1988)
H636C	H636 complemented strain with	This study
	a copy of *oprF* downstream	
	pBAD promoter	
**Plasmids**
pRW5	OprF cloned in pUCP19 vector, Cb^r^	Woodruff and Hancock (1988)
pJN105	araC-pBAD cassette cloned in pBBR1MCS-5,	Newman and Fuqua (1999)
	Gm^r^	
pJN2133	PA2133 cloned in pJN105, Gm^r^	Hickman et al. (2005)
mini-CTX1	Plasmid for the integration of genes into	Hoang et al. (2000)
	the *att* site of the *P. aeruginosa* chromosome, Tc^r^	
mini-CTX-	araC-pBAD cassette cloned in mini-CTX1, Tc^r^	This study
araC-pBAD
mini-CTX-	oprF cloned upstream pBAD promoter in	
araC-pBAD-oprF	mini-CTX-araC-pBAD, Tc^r^	This study
PcdrA-gfp		Rybtke et al. (2012)

Cb^r^, carbenicillin resistance; Gm^r^, gentamycine resistance; Tc^r^, tetracycline resistance.

### EPS Production Assays

To observe colony morphology, overnight cultures were diluted at an OD_580_ of 0.08 in LB (8.10^7^ CFU.ml**^-^**^1^) and 5 μl were spotted onto a LB plate containing 40 μg.ml**^-^**^1^ of CR and 20 μg Coomassie brilliant blue as described by Friedman and Kolter (2004a,b). Plates were incubated at 37°C overnight before visual inspection of the colony morphology. To quantify the CR binding on bacterial cells, a CR release assay was achieved as previously described (Lee et al., 2007). Briefly, an aliquot of overnight cultures corresponding to 10^9^ cells, were centrifuged at 7,500 *g* during 10 min at room temperature. Cells were resuspended in 1 ml of LB containing CR (40 μg.ml**^-^**^1^), and incubated for 15 min at room temperature before centrifugation at 7,500 *g* during 10 min. The absorbance of the unbound CR was measured in the supernatant at 490 nm.

### Total Carbohydrate Content Assay

Sugar composition of the cell-associated carbohydrates was determined by gas chromatography analysis of trimethylsilyl methyl glycoside derivatives using inositol as internal standard for quantification. Bacteria (200 ml), grown overnight in LB medium, were centrifuged at 7,500 *g* for 5 min. Cells were washed gently with 10 ml of distilled water before centrifugation at 7,500 *g* for 5 min. EPS were detached from cells as followed. Ten ml of distilled water was added to the cell pellet, and the tubes were vigorously mixed each 2 min for 14 min before centrifugation at 7,500 *g* for 10 min. This step was repeated five times. The supernatants were collected, pooled, filtered through a 0.2 μm filter and precipitated overnight at -20°C by adding cold ethanol to a final concentration of 70% (v/v). The precipitate was collected by centrifugation at 13,000 *g* for 1h at 4°C, resuspended in 5 ml of distilled water, dialyzed against distilled water using a 6–8 kDa molecular weight cut-off membrane for 24 h at 4°C, and lyophilisated. Samples (about 2 mg of dried material) and 50 μL of 2.10**^-^**^3^M inositol were hydrolyzed in 0.5 mL of 2 M trifluoroacetic acid for 2 h at 110°C, dried and then heated in 0.250 mL of dry 1 M HCl in methanol at 80°C for 24 h for methanolysis. After evaporation of the methanol, the sample was resuspended in 500 μL of methanol and dried again. The methyl glycosides were then converted into their trimethylsilyl derivatives by heating the samples for 20 min at 80°C in 200 μL of hexamethyldisilazane/trimethylchlorosilane/pyridine: 3/1/9. After evaporation of the reagent, the samples were suspended in 100 μL of cyclohexane before being injected on a FDB-1 column (DB-1 Supelco). Chromatographic data were integrated with gas chromatography Star Workstation software (Varian), each surface being corrected according to its response factor. For comparison of amounts of cell-associated carbohydrates, total monosaccharide contents for each sample were normalized according to the OD measured at 580 nm.

### General DNA Procedures

Restriction enzymes, T4 DNA ligase, and alkaline phosphatase were purchased from New England Biolabs (Ipswich, MA, USA) and used accordingly to the manufacturer. PCR assays were carried out with 1 μg of *P. aeruginosa* chromosomal DNA, 20 pmol of each primer and failsafe Taq polymerase (Epicentre Biotechnologies, Madison, WI, USA). When necessary, PCR products and plasmids were purified with the QIAquick or QIAprep Spin Miniprep kits (QIAGEN), respectively. DNA sequencing was achieved by Genome Express (France). *E. coli* [commercial electrocompetent DH5α cells (Promega, Madison, WI, USA) or S17.1 cells] and *P. aeruginosa* cells were transformed by electroporation or by conjugation as previously described (Bouffartigues et al., 2012).

### H636C Complemented Strain Construction

To complement the H636 *oprF* mutant strain with a copy of the *oprF* gene into the chromosome, the mini-CTX1-araC-pBAD was constructed as follows. The region containing *araC* and the pBAD promoter was amplified from pJN105 (**Table 1**) using the primer pair FaraC-pBAD and RaraC-pBAD (**Table 2**) and cloned into pCR2.1-TA (Invitrogen, Carlsbad, CA, USA). Insert was then cut with *Sac*I and *Nhe*I restriction enzymes and inserted into mini-CTX1 using the *Sac*I and *Spe*I sites. A 1113 bp DNA fragment containing the *oprF* gene (PA1777) was amplified by PCR using the primer pair FoprFPstI and RoprFHindIII (**Table 2**). The PCR product was digested with *Pst*I and *Hind*III and ligated into the *Pst*I-*Hind*III digested mini-CTX1-araC-pBAD vector to create the mini-CTX1-araC-pBAD-oprF. The sequence of this construct was verified by DNA sequencing. This vector was constructed in DH5α, purified and transferred into *E. coli* SM10 strain. The mini-CTX1-araC-pBAD-oprF vector was mobilized from *E. coli* SM10 into H636 by conjugation. Transconjugants were selected onto PIA agar plate containing 250 μg.ml^-1^ of tetracyclin. The insertion was verified by PCR using the FoprFPstI and RoprFHindIII primers. It is known that the pBAD promoter is not fully locked in *P. aeruginosa*, we used the basal level of expression from this promoter, without addition of arabinose, to obtain adequate amount of OprF in complementation experiments (Becher and Schweizer, 2000; Hoang et al., 2000).

**Table 2:** Primer sequences of the indicated genes used for quantitative RT-PCR reactions and for the construction of the mini-CTX1-araC-pBAD and its derivative vector mini-CTX1-araC-pBAD-oprF.

PA number	Gene name	Primer name	Sequence (5′-3′)
PA2063	*pelB*	FPA3063	CGGCTACGTGCAGCGTTAT
		RPA3063	CACTGCATGCGTTCCTTGAC
PA2231	*pslB*	FPA2231	ACACCAACGAATCCACCTTCA
		RPA2231	CGCTCTGTACCTCGATCATCAC
PA3621.1	*rsmZ*	FPA3621.1	CGTACAGGGAACACGCAAC
		RPA3621.1	ATTACCCCGCCCACTCTTC
PA0527.1	*rsmY*	FPA0527.1	CGCCAAAGACAATACGGAAAC
		RPA0527.1	TTTTGCAGACCTCTATCCTGACATC
PA1776	*sigX*	FPA1776	AATTGATGCGGCGTTACCA
		RPA1776	CCAGGTAGCGGGCACAGA
PA1775	*cmpX*	FPA1775	GGCAGATCATTGCAGGAATCTAC
		RPA1775	TCTCTTCAATAGTGCCTTCAACGT
PA0762	*algU*	FPA0762	GAAGCCCGAGTCTATCTTGG
		RPA0762	GCGATACCTCTCTTGGCATT
PA3540	*algD*	FPA3540	GGGCTATGTCGGTGCAGTATG
		RPA3540	GCGACTTGCCCTGGTTGAT
PA4843	*PA4843*	FPA4843	CCTGGGCACCGAATTGG
		RPA4843	CGGCGGACAGGTAGATGATC
PA2072	*PA2072*	FPA2072	CCAGGCATCAGGACGACAT
		RPA2072	CGATTCTGCAGCGCCTTT
PA1181	*PA1181*	FPA1181	CCAGATGGAGAAGCGCTACCTCGCTT
		RPA1181	CGCTTGCGACTGTCGATATC
16sRNA		F16SRNA	AACCTGGGAACTGCATCCAA
		R16SRNA	CTTCGCCACTGGTGTTCCTT
PA1777	*oprF*	FoprFPstI	^∗^taataactgcagAGATGGGGATTTAACG
		RoprFHindIII	^∗^taataaaagcttTCCTTAGAGGCTCAGCCGATT
		FaraC-PBAD	^∗^aacatatgCGTCAATTGTCTGATTCGTTACCAAT
		RaraC-PBAD	^∗^aatcgctagcCCAAAAAAACGG

^∗^Nucleotides not included in the gene sequence are indicated in lower case.

### Quantitative RT-PCR

Extraction of RNAs, synthesis of cDNAs and real time PCR were achieved as previously described (Gicquel et al., 2013), using primers described in **Table 2**. PCR reactions were performed in triplicate and the standard deviations were lower than 0.15 CT. The relative quantification of the mRNAs or sRNAs of interest was obtained by the comparative CT (2^-ΔΔCt^) method (Livak and Schmittgen, 2001), using 16S rRNA as endogenous control (Corbella and Puyet, 2003).

### c-di-GMP Quantification

Indirect c-di-GMP quantification was evaluated using P_cdrA_-*gfp* plasmid, in which the c-di-GMP depending *cdrA* promoter is fused to *gfp* (Rybtke et al., 2012). *P. aeruginosa* strains containing the P_cdrA_-*gfp* plasmid were grown overnight and subcultured in LB with gentamicin to a starting OD_600_ of 0.1 (10^8^ CFU.ml^-1^). After 4 h incubation at 37°C in shaking conditions, 1 mL of culture was harvested and re-suspended in the same volume of 1x PBS. OD_600_ and fluorescence (excitation 485 nm, emission 520 nm) was measured in a black 96-well plate with see-through flat bottom (Falcon) using a FLUOstarOptima plate reader (BMG Labtech). Quantifications were performed in triplicate and data are presented as relative fluorescent units (RFUs) which are arbitrary fluorescent units corrected for cell density.

c-di-GMP was also directly quantified by LC-MS/MS. Extraction and quantification of intracellular c-di-GMP was performed with some modifications as previously described (Spangler et al., 2010; Strehmel et al., 2015). Cultures of *P. aeruginosa* were grown for 4 h in LB medium at 37°C under shaking conditions to an optical density OD_600_ of ∼2.0 (2.10^9^ CFU.ml^-1^). Subsequent aliquots of 10 ml of each culture, adjusted to a cell density OD_600_ of 0.5 (5.10^8^ CFU.ml^-1^) by dilution with LB medium, were harvested by centrifugation for 2 min, 8000 × *g* and 4°C. Cell pellets were resuspended in 600 μl ice-cold extraction buffer [40% (v/v) acetonitrile, 40% (vol/vol) methanol and 20% (vol/vol) ddH_2_O] by vigorous vortexing with and without 5 μl of a 10 μg.ml^-1^ solution of xanthosine-(3′,5′)-cyclic monophosphate (cXMP; BioLog Life Science Institute; Bremen, Germany) in ddH_2_O as an internal standard. After 15 min of incubation on ice, cells were lysed at 95°C for 10 min, followed by centrifugation at 20000 × *g* and 4°C for 5 min. Supernatants were collected in a new 1.5 ml reaction tube and stored on ice. Remaining pellets were again resuspended in 400 μl extraction buffer, incubated on ice for 15 min and subjected to centrifugation (15000 × *g*, 4°C, 5 min). This procedure was repeated, and supernatants of all three extraction steps were pooled and subsequently evaporated using a concentrator 5301 (Eppendorf, Hamburg, Germany) at 45°C. The remaining residue were then resuspended in 500 μl ddH_2_O and c-di-GMP levels were quantified by LC-MS/MS as described previously (Strehmel et al., 2015), with the following modifications. The chromatographic separation was performed on a 1100 Series HPLC system (Agilent, Waldbronn, Germany) using a Multospher AQ RP 18, 5 μm, 250 mm× 4.0 mm HPLC column (CS Chromatography Service GmbH, Langerwehe, Germany) in a gradient mode using 10 mM ammonium acetate with 0.1% acetic acid as eluent A and methanol as eluent B. The injection volume of each sample was set to 40 μl and the flow rate was 0.4 ml/min. The gradient program was as follows: from 0 to 4 min 100% A, followed by a linear gradient from 100 to 80% A in 1 min, held for 2 min at 80% A, followed by a linear gradient from 80 to 60% A in 1 min and held for additional 9 min at 60%. Finally, re-equilibration of the column was obtained by constantly running 100% A for 16 min.

Electrospray ionization (ESI) MS was performed on an API 4000 triple quadrupole mass spectrometer (Applied Biosystems, Toronto, ON, Canada) using a turbo ion spray interface used in *positive mode* at an ionization potential of 5000 V and a temperature of 400°C using nitrogen as curtain, nebulizer, heater and collision gas. The parameter settings were optimized by infusion experiments. Data were acquired in multiple reaction monitoring (MRM) mode using the Analyst software version 1.6. (Applied Biosystems, Toronto, ON, Canada). Identification and quantification of c-di-GMP was performed by using three specific mass transitions from molecule ion m/z 691 to the product ions m/z 152, m/z 135, and m/z 540. The external calibration was carried out at c-di-GMP concentrations ranging from 10 to 200 ng in 500 μl ddH_2_O using the internal standard cXMP (50 ng). Obtained concentrations of c-di-GMP were normalized against total protein contents of respective cultures, which were determined by the bicinchoninic acid assay (Smith et al., 1985). All experiments were performed for three independent cultures each analyzed in duplicate.

### Bacterial Attachment Assay on Glass and Scanning Electron Microscopy

Bacteria were grown overnight in LB at 37°C, harvested by centrifugation for 10 min at 7,000 *g*, washed twice, and suspended in 0.9% NaCl to an OD_600_ of 0.6 (6.10^8^ CFU.ml^-1^). Twenty-five ml were then poured into a Petri dish containing a glass slide (24 × 50 mm) and stored at 37°C for 2 h. The slide was then washed for 5 min with sterile demineralized water to remove non-adherent cells. The attached cells were observed with a scanning electron microscope (SEM) as follows. Each sample was fixed with 3% glutaraldehyde overnight at 4°C and washed three times with 0.1 M phosphate buffer (pH 7.35). The cells were then sequentially dehydrated with 70, 95, and 100% ethanol (three times for 10 min each). The samples were dried in ethanol in a CPD 030 Critical Point Dryer (Bal-Tec, France), using CO_2_ as a transitional fluid to reach the critical point. The samples were mounted on aluminum stubs and coated for 120 s at 20 mA with gold/palladium alloy using a 501 sputter coater (Edwards Pirani, UK) and observed with a JEOL 6460LV microscope (JEOL Ltd, Japan) at a magnification of 4,500. The voltage was kept at 10 or 15 kV, at an average distance from the electron gun of about 10 mm.

### Biofilm Formation in a Flow Cell System and Confocal Laser Scanning Microscopy

Biofilms were grown at 37°C in a three-channel flow cell with individual channel dimensions of 1 mm× 4 mm × 40 mm (Biocentrum, DTU, Danemark, Pamp et al., 2009), using a microscope coverslip (ST1, VWR) as substratum. The flow system was assembled, prepared and sterilized as described by Tolker-Nielsen and Sternberg (2011). Flow cells were sterilized by flushing 0.5% sodium hypochlorite for 30 min, then rinsed overnight with sterile water using a 250S Watson Marlow peristaltic pump. Bacteria were grown in LB, harvested by centrifugation for 10 min at 7,000 *g*, washed twice and suspended in 0.9% NaCl to obtain an OD_600_ of 0.6. Each channel was inoculated with 1 ml of bacterial suspension and left for attachment during 2 h without flow at 37°C. LB medium was then pumped with a 2.5 ml h^-1^ flow. Biofilms were visualized after 24 and 48 h by confocal laser scanning microscopy (CLSM) with a TCS-SP2 microscope (Leica Microsystems, Heidelberg, Germany), using a 63x oil immersion objective. Image stacks were collected and processed using Leica Confocal Software and Adobe Photoshop. The thicknesses (μm) and biovolumes (in μm^3^/μm^2^) of the biofilms were measured using the COMSTAT software (Heydorn et al., 2000). Each experiment was made in triplicate and 10 positions were analyzed per channel.

### Biofilm Formation on Polystyrene and Crystal Violet Staining

Quantification of biofilm formation was performed in 12-wells polystyrene microtiter plates as previously described (Bouffartigues et al., 2014). Briefly, LB medium (2 ml per well) supplemented with appropriate antibiotics was inoculated to a final OD_580_ of 0.08 (8.10^7^ CFU.ml^-1^) and incubated at 37°C without shaking for 24 h. Biofilms were stained with 3 ml of 0.4% of crystal violet (CV) for 20 min at room temperature, and washed with 3 ml of water until removing the unbound dye. After washing, CV was solubilized in 100% ethanol before measuring the absorbance at 595 nm. For each biofilm assay, three independent experimental repetitions were performed.

## Results

### The Lack of OprF Results in Increased EPS Production, Cell Aggregation and Biofilm Formation

**FIGURE 1:**
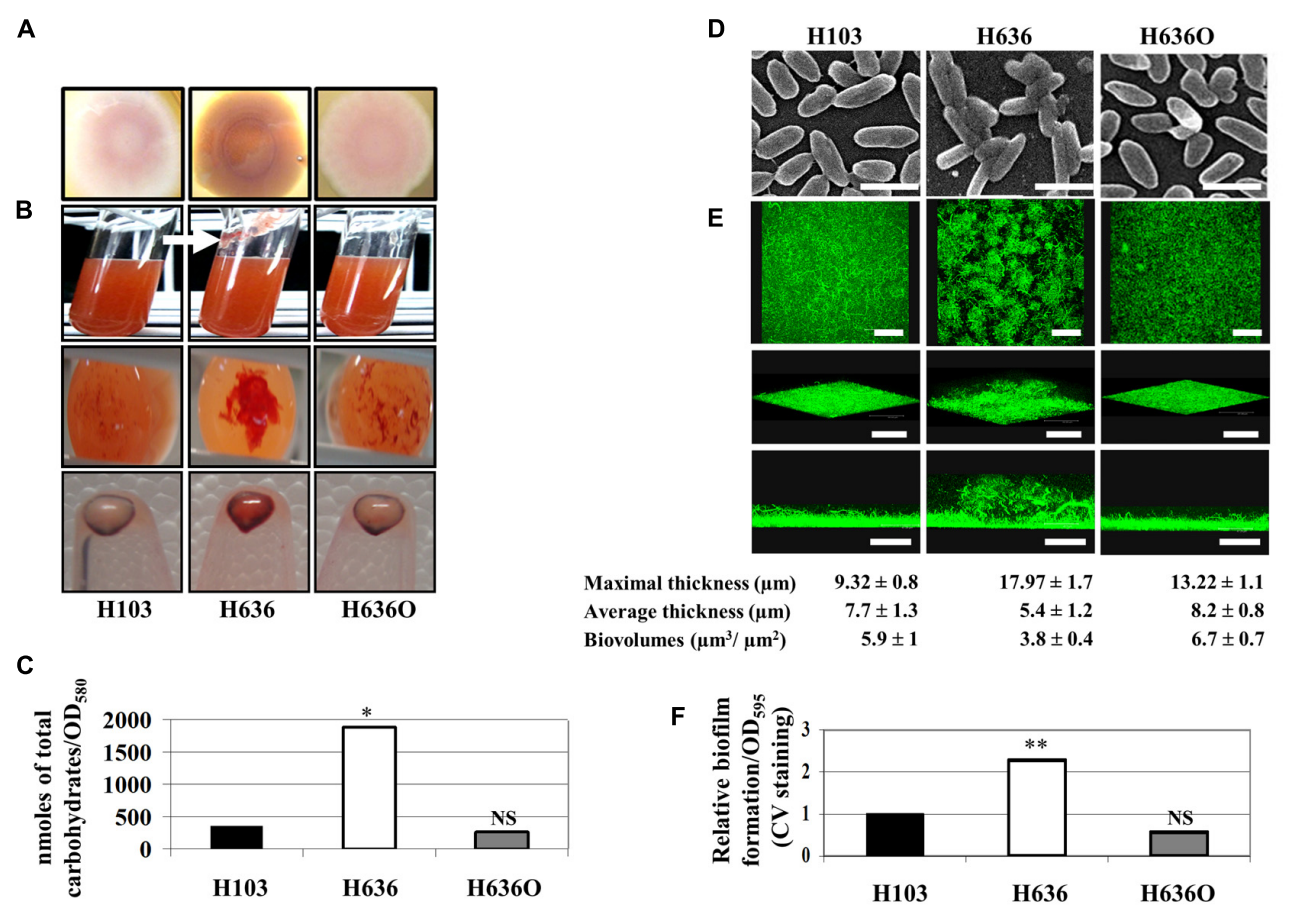
**The absence of OprF led to increase EPS production and biofilm formation in *P. aeruginosa*. (A)** Colony morphology observed on Congo red (CR) containing LB agar plates. **(B)** H103, H636, and H636O were grown in CR containing LB for 24 h at 37°C. Top images: a slime production is indicated by a white arrow in case of H636; middle images: CR colored aggregates were observed at the bottom of the cultures; bottom images: CR binding of pelleted cells (10^9^). **(C)** Cell-associated carbohydrates were quantified by gas chromatography. Statistics were achieved by unpaired *t*-test. ^∗^*p* < 0.05, NS, not significant. **(D)** Scanning electron microscopy images of H103, H636, and H636O allowed to attach onto a glass coverslip for 2 h (enlargement: 4,500 x; scales represent 2 μm). **(E)** Biofilms of H103, H636, and H636O grown in flow cells for 24 h and examined by CLSM. Top images, top views (x, y-plane; scales represent 48 μm); intermediate images, cross section views (scales represent 67 μm); bottom images, 3D-modelizations (x, y, z-axes, scales represent 48 μm). Maximal (μm), average thicknesses (μm), and biovolumes (μm^3^/μm^2^) were determined by COMSTAT analyses. The averages and standard deviations were calculated from 10 samples. **(F)** Microtiter grown biofilm formed by H103, H636, and H636O. Biofilms were quantified by measuring absorbance at 595 nm after crystal violet (CV) staining. At least six assays were performed for each strain. Statistics were achieved by unpaired *t*-test. ^∗∗^*p* < 0.01, NS, not significant.

We investigated the role of OprF in biofilm formation and EPS production by comparing three strains, i.e., the *P. aeruginosa* H103 wild-type strain (a PAO1 derivative, Hancock and Carey, 1979), the isogenic *oprF* mutant H636 (Woodruff and Hancock, 1988), and the *oprF* complemented control strain H636O (Woodruff and Hancock, 1988). We first examined colony morphologies on CR plates at 37°C. As shown in **Figure 1A**, the *P. aeruginosa* H636 colony, i.e., the *oprF* mutant, displayed a marked red staining, suggesting higher polysaccharide production when compared to the H103 wild-type strain. Upon complementation of the *oprF* mutation, a lower polysaccharide production was restored, confirming that the phenotype was due to the *oprF* deletion. Accordingly, when strains were grown in liquid LB medium, CR staining revealed the presence of cell aggregates and slime attached to the glass in H636, but not in H103 or H636O (**Figure 1B**). Upon centrifugation of the bacterial cultures, the cell pellets showed higher CR staining for H636 but not for H103 or H636O (**Figure 1B**). Quantification of the total cell-associated carbohydrates by gas chromatography confirmed that the levels of EPS in the H636 *oprF* mutant are fivefold higher than in H103 or H636O strains (**Figure 1C**).

It is well established that increased EPS production may result in cell aggregation and biofilm formation. Bacteria were thus allowed to attach onto a glass coverslip for 2 h and then observed by scanning electron microscopy (SEM). SEM images showed that H636 cells were slightly thinner than H103 and H636O, and cells aggregates could be observed, suggesting that the absence of OprF led to cell surface alterations that favored interactions between bacteria (**Figure 1D**). In addition, biofilm formation in flow cells was examined by CLSM after 24 h growth. As shown in **Figure 1E**, H103 and H636O biofilms were homogeneous and flat, displaying similar thicknesses and biovolumes. Conversely, the H636 *oprF* mutant formed a biofilm with heterogeneous cell distribution, increased maximal thickness and weaker biovolume. CV staining and quantification of biofilms grown in microtitre plates revealed that the *oprF* mutant strain yielded a 2.3-fold higher biofilm biomass than the other two strains (**Figure 1F**).

### The OprF-Deficient Strain Displays Increased Levels of *pel* mRNA and c-di-GMP

**FIGURE 2:**
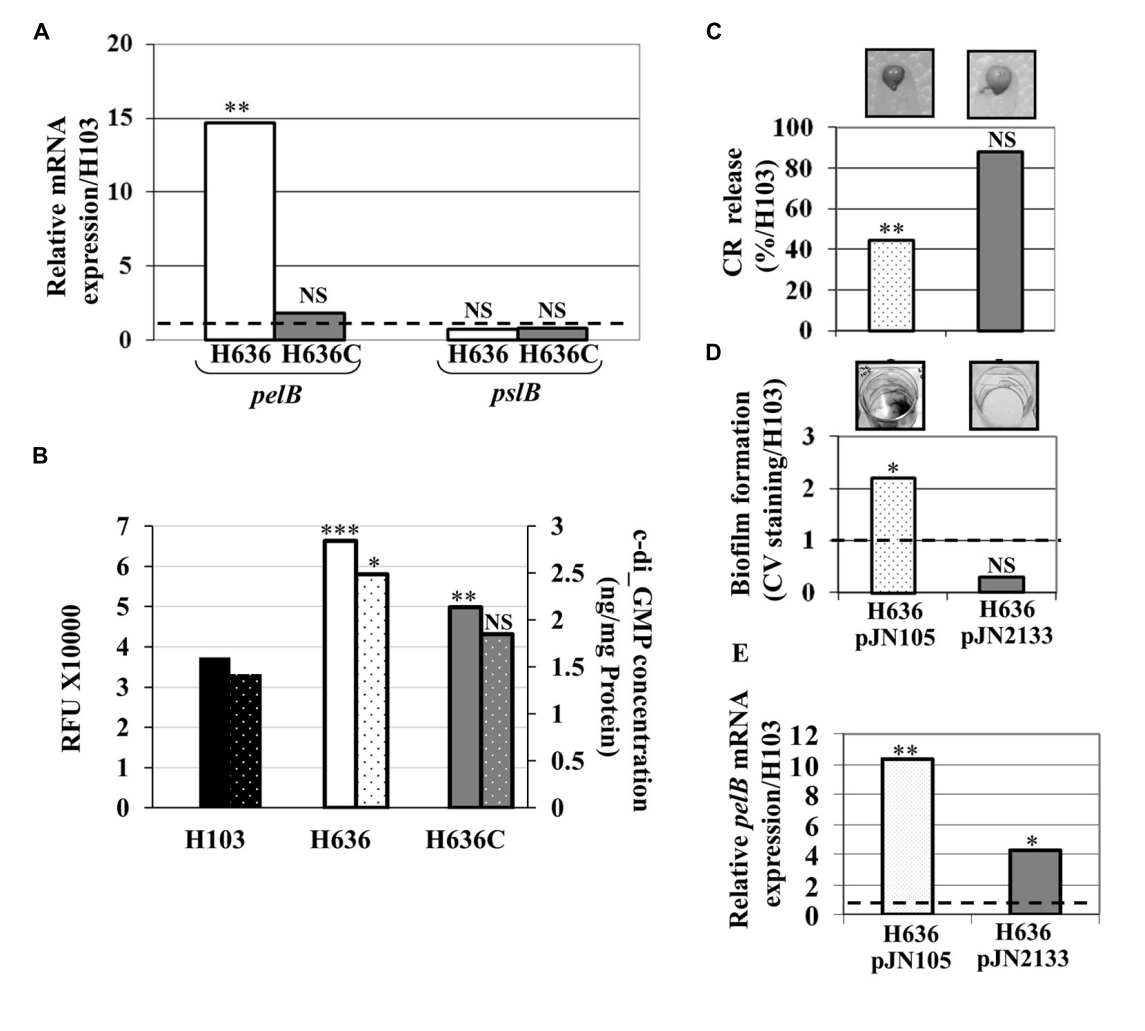
**The c-di-GMP level is increased in the *oprF* mutant. (A)** Relative *pel* and *psl* mRNA expression in H636 (white bars) and H636C (gray bars) relatively to H103 (dashed black line). **(B)** c-di-GMP level evaluation using the P_cdrA_-*gfp* reporter fusion (full colored bars), and by LC/MS/MS quantification (dotted colored bars), harbored by H103 (black bar), H636 *oprF* mutant (white bar) and the complemented H636C strain (gray bar). **(C)** Exopolysaccharide production by H636 harboring pJN105 (dotted white bar) or pJN2133 (gray bar), using the CR release assay. 100% correspond to the EPS production by *P. aeruginosa* H103. **(D)** Biofilms formed by H636 harboring pJN105 (dotted white bar) or pJN2133 (gry bar) were quantified relatively to H103 by CV staining. **(E)** qRT-PCR assays of *pelB* mRNA level in H636 harboring pJN105 (dotted white bars) or pJN2133 (gray bars), relatively to H103. Each experiment **(A–E)** was performed at least three times. Statistics were achieved by unpaired *t*-test: ^∗^*p* < 0.05, ^∗∗^*p* < 0.01, ^∗∗∗^*p* < 0.001, NS, not significant, relatively to H103.

Since Psl and Pel are the major EPS of *P. aeruginosa* PAO1 biofilm matrix (Hickman et al., 2005), we quantified by qRT-PCR the levels of the *pelB* and *pslB* transcripts. While *pslB* mRNA levels were unaffected by the *oprF* mutation, expression of *pelB* was 15-fold up-regulated in the H636 mutant strain (**Figure 2A**). Complementation of the mutant restored the expression of *pelB* to wild-type level, confirming that the observed variation was OprF-dependent. Since the expression of *pel* and *psl* is stimulated by high intracellular c-di-GMP levels (Lee et al., 2007; Starkey et al., 2009; Borlee et al., 2010), the latter was tested using the P_cdrA_-*gfp* reporter system in which *gfp* is fused to the c-di-GMP-dependent *cdrA* (Rybtke et al., 2012). The reporter plasmid was introduced into the wild-type H103, the H636 mutant strain, and the *oprF* complemented strain H636C. In this case and in the subsequent experiments, complementation was achieved from a single copy of *oprF* inserted in the chromosome (see Materials and Methods). The **Figure 2B** shows that the activity of the *cdrA* promoter was *ca* 1.8-fold higher in H636 than in H103, a phenotype that was partly complemented in H636C. This result was confirmed by direct quantification of intracellular c-di-GMP (**Figure 2B**) using LC-MS/MS (see Materials and Methods).

To assess whether we could revert the *oprF* mutant phenotypes by modulating the c-di-GMP level, the pJN2133 plasmid carrying the gene encoding the phosphodiesterase PA2133 under control of the pBAD promoter (Hickman et al., 2005), was introduced in the H636 mutant strain. Basal expression of PA2133 without arabinose induction led to restore CR binding (**Figure 2C**) and biofilm formation (**Figure 2D**) to wild-type levels in the pJN2133-carrying strain. In agreement with this observation, we observed that reducing the c-di-GMP level led to a significant decrease in *pelB* expression (**Figure 2E**). Noteworthy, the level of *pelB* was not fully restored, suggesting that additional regulatory mechanisms may account for the up-regulation of the *pel* genes in the *oprF* mutant (**Figure 2E**).

### *rsmZ* is Up-Regulated in the *oprF* Mutant

**FIGURE 3:**
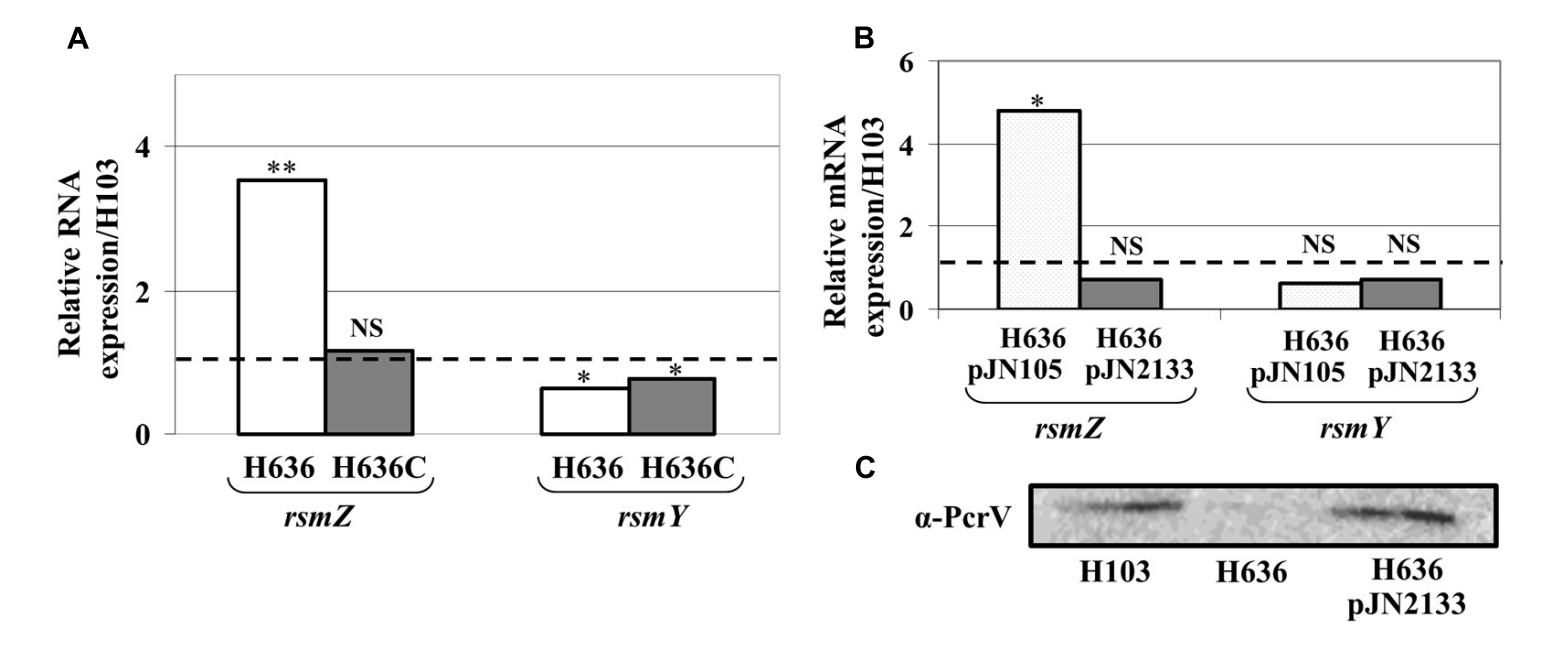
**The lack of OprF led to increase *rsmZ*, but not *rsmY* expression. (A)** Relative expression of the small RNAs *rsmZ* and *rsmY* in H636 mutant (white bars) and the complemented strain H636C (gray bars), relatively to H103 (dashed black line). **(B)** Relative *rsmZ* and *rsmY* sRNA levels in H636 mutant strain harboring pJN105 (dotted white bars) or pJN2133 (gray bars), relatively to H103 (dashed black line). **(C)** Western blot analyses of T3SS α-PcrV in *P. aeruginosa* H103, H636, and H636 harboring pJN2133. Each experiment was performed at least three times. Statistics were done by unpaired *t*-test. ^∗^*p* < 0.05, ^∗∗^*p* < 0.01, NS, not significant.

Pel production is known to be post-transcriptionally repressed by the RNA binding protein RsmA, a downstream target of the GacS/GacA two-component system (TCS; Brencic and Lory, 2009; Irie et al., 2010). This regulatory system positively controls factors involved in biofilm formation through the production of two small non-coding RNAs, *rsmY* and *rsmZ* that sequester RsmA and thus interfere with its function (Gooderham and Hancock, 2009; Mikkelsen et al., 2011). Using qRT-PCR experiments, we investigated whether the production of *rsmY* and *rsmZ* was affected in the H636 *oprF* mutant. As shown in **Figure 3A**, *rsmZ* expression was increased by about 3.5-fold, while that of *rsmY* was slightly decreased in the *oprF* mutant. Complementation of the mutant led to restoration of *rsmZ* levels. Reducing the c-di-GMP level by overexpressing PA2133 led to lower *rsmZ* expression without affecting that of *rsmY*, suggesting that *rsmZ* transcription is connected to the c-di-GMP increase in the *oprF* mutant (**Figure 3B**). Finally, we have previously shown that the H636 *oprF* mutant was impaired in the production of components of the type III secretion system (T3SS; Fito-Boncompte et al., 2011), which is also regulated by the Gac/RsmA signaling network. Consistently, the production of the T3SS needle tip, PcrV, was strongly reduced in H636 compared to H103. The levels of PcrV were restored when the *oprF* mutation was compensated by reducing the c-di-GMP levels in H636 (**Figure 3C**).

### The ECF Sigma Factors AlgU and SigX are Up-Regulated in the *oprF* Mutant

**FIGURE 4:**
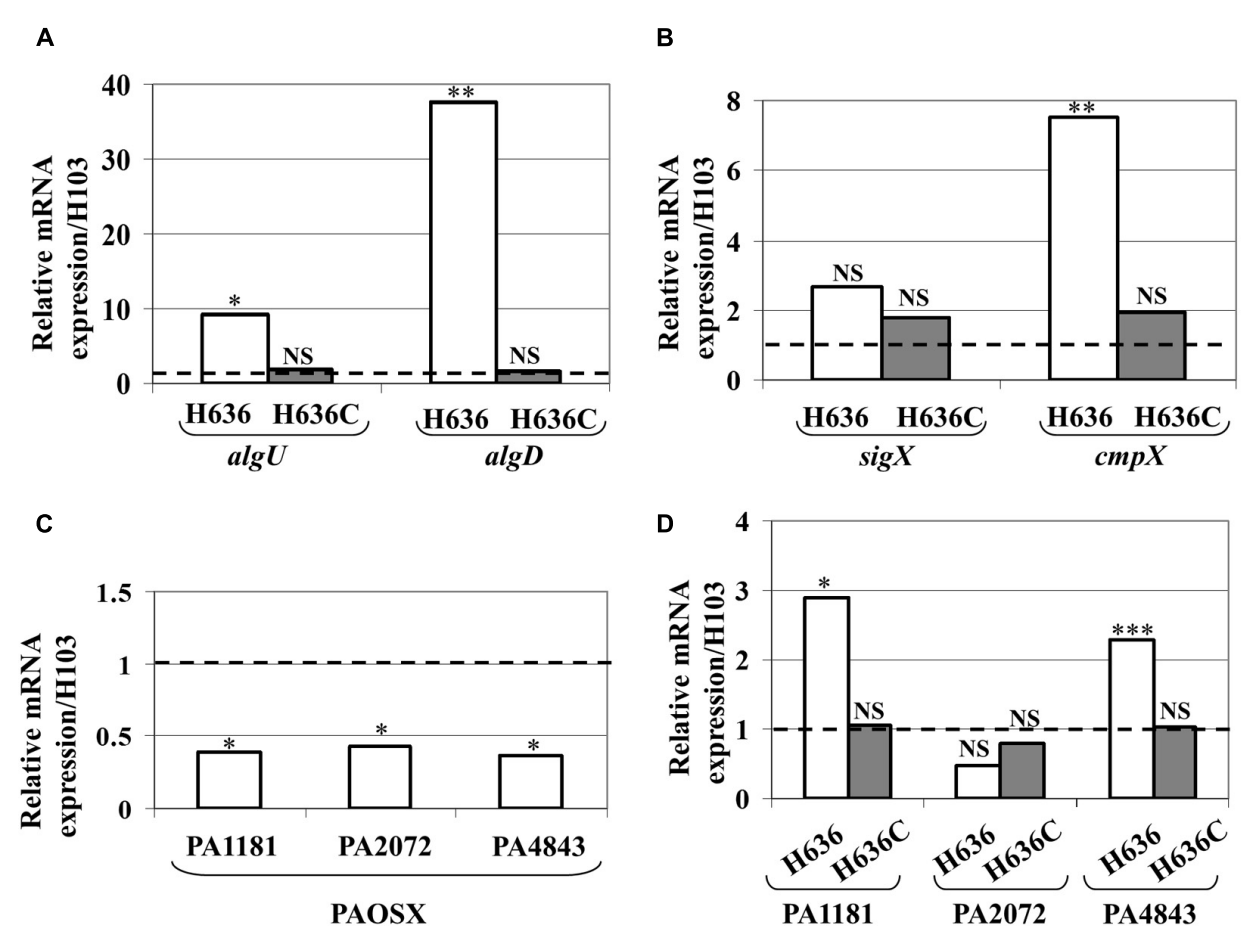
**Increased expression and activity of the extracytoplasmic function sigma factors AlgU and SigX.** Relative mRNA levels of *algU* and *algD* **(A)**, *sigX* and *cmpX* **(B)** in *P. aeruginosa* H636 (white bars) and H636C (gray bars) relatively to H103. **(C)** Relative mRNA levels of PA1181, PA2072 and *adcA* in PAOSX [*sigX* mutant strain (Bouffartigues et al., 2012) relatively to H103]. **(D)** Relative mRNA levels of the SigX-dependent PA1181, PA2072 and *adcA* (PA4843) gene in *P. aeruginosa* H636 (white bars) and H636C (gray bars) relatively to H103. Each experiment was performed at least three times. Statistics were achieved by unpaired *t*-test. ^∗^*p* < 0.05, ^∗∗^*p* < 0.01, ^∗∗∗^*p* < 0.001, NS, not significant.

OprF was suspected to be involved in cell shape maintenance, partly through connecting the OM with the peptidoglycan layer. It has been suggested that the absence of OprF leads to alterations of the cell wall integrity (Woodruff and Hancock, 1989; Rawling et al., 1998). *P. aeruginosa* possesses two extracytoplasmic sigma factors involved in maintaining cell wall integrity, the RpoE homologue AlgU (Wood and Ohman, 2009) and SigX (Boechat et al., 2013; Gicquel et al., 2013; Blanka et al., 2014). As shown in **Figure 4A**, the expression of *algU* and *algD* genes, which are direct AlgU targets (Schurr et al., 1993), was strongly increased in the *oprF* mutant, a phenotype that is restored in the H636C strain. This strongly supports the idea that the *oprF* mutant strain encounters cell wall stress. On the contrary, the expression of *sigX* was not significantly altered in the conditions tested. However, the expression of *cmpX*, a direct target of SigX (Bouffartigues et al., 2012; Gicquel et al., 2013; Blanka et al., 2014), was significantly increased in the *oprF* mutant (**Figure 4B**). We thus hypothesized that SigX activity level is higher in H636 and examined the expression of genes from the SigX regulon that are involved in c-di-GMP metabolism, i.e., PA1181, PA2072 and *adcA* (PA4843; Gicquel et al., 2013; Blanka et al., 2014). As shown in **Figure 4C**, PA1181, PA2072 and *adcA* (PA4843) are down-regulated in the isogenic *sigX* mutant of *P. aeruginosa* H103, confirming previous observations made on strain PA14 (Blanka et al., 2014). Using qRT-PCR, we observed that PA1181 and *adcA* mRNAs are overproduced in H636 (but not in the complemented strain) by 2.9- and 2.1-fold, respectively. In contrast, the expression of PA2072 was unaffected (**Figure 4D**). Since AdcA has been shown to have diguanylate cyclase activity (Jones et al., 2014), and PA1181 contains both GGDEF and EAL motifs, our data suggest that the two proteins may be responsible for the c-di-GMP increase in the *oprF* mutant strain.

## Discussion

In a previous study, we have shown that an *oprF* mutant is altered in the production of many virulence factors, including pyocyanin, elastase and T3SS effectors (Fito-Boncompte et al., 2011). Here, we further show that an *oprF* mutant overproduces polysaccharides, a phenotype that is mainly linked to *pel* gene overexpression rather than *psl*. In PA14, or when overexpressed in PAO1, Pel promotes attachment to abiotic surfaces, aggregation in broth culture, and biofilm formation (Yang et al., 2011; Colvin et al., 2012). Accordingly, the *oprF* mutant displayed an aggregative phenotype and an increased biofilm formation in our conditions. Interestingly, the biofilm structure produced by the mutant strain was altered. The maximal thickness was increased while the biofilm biovolume was reduced. These data suggest that H636 cells grew slower than both the wild-type and the complemented mutant strains. Otherwise, the lack of OprF may weaken the cell structure leading to increased bacterial death within the biofilm. We also observed increased c-di-GMP level in the *oprF* mutant. High levels of c-di-GMP are known to (i) impact positively polysaccharide production and biofilm formation (Hickman et al., 2005; Güvener and Harwood, 2007; Starkey et al., 2009; Borlee et al., 2010), and (ii) impact negatively the production of T3SS components (Moscoso et al., 2011), which we were able to confirm in the *oprF* mutant. Decrease of the c-di-GMP level in the *oprF* mutant strain upon overproduction of a phosphodiesterase led to the restoration of polysaccharide production, biofilm formation and T3SS activity to wild-type levels. These observations suggest that an elevated c-di-GMP level could be the leading cause of the phenotypic alterations in this strain. Both *pel* and *psl* genes are known to be regulated by intracellular c-di-GMP level (Hickman et al., 2005), so the question remains on how c-di-GMP increase in the *oprF* mutant specifically affects Pel synthesis but not Psl. Recently, another study has shown that c-di-GMP produced by the diguanylate cyclase WspR specifically affects Pel but not Psl synthesis (Huangyutitham et al., 2013). This same study suggests that phenotypes that are very dominant in *P. aeruginosa* may not be much affected by the small c-di-GMP increase [this could be the case for Psl, which is the EPS that is mostly responsible for attachment and biofilm formation in *P. aeruginosa* strain PAO1 (Colvin et al., 2012)]. This study also suggests that the differential effects are due to differences in the affinity of the receptor proteins for c-di-GMP. PelD and FleQ are the two proteins that regulate Pel synthesis in response to c-di-GMP and their affinity to c-di-GMP is in the micromolar range (lower affinity; Hickman et al., 2005; Whitney et al., 2012), whereas other known c-di-GMP receptors have affinities in the nanomolar range (higher affinity; Christen et al., 2010). So it is conceivable that a change in the c-di-GMP level like the one observed in the *oprF* mutant could affect FleQ and/or PelD activity but not other high-affinity receptors which are already saturated for c-di-GMP binding. Finally, the levels of *pelB* mRNA were not fully reverted to wild-type levels upon artificial reduction of the c-di-GMP level in the *oprF* mutant, suggesting that factors other than c-di-GMP may contribute to *pelB* increased expression in the *oprF* mutant.

It is known that the *pel* and *psl* mRNAs are furthermore post-transcriptionally repressed by RsmA, the activity of which is controlled by the two sRNAs *rsmZ* and *rsmY* through complex regulatory systems involving several sensor kinases and accessory components (Mikkelsen et al., 2011). Interestingly, we show that *rsmZ* transcription is increased in response to the higher c-di-GMP level displayed by the *oprF* mutant. Expression of these sRNAs is positively regulated by the phosphorylated GacA response regulator from the TCS GacS/GacA. GacS is itself regulated by the orphan sensors, LadS and RetS, acting positively and negatively on GacA, respectively. PhoP, the regulator of the PhoPQ TCS that is activated in response to low magnesium concentration, also controls expression of *rsmZ* but not *rsmY* (Mulcahy and Lewenza, 2011). Noticeably, the PA1181 gene encoding a c-di-GMP cyclase is located nearby PA1179 and PA1180, encoding the PhoPQ TCS. However, the three genes were not predicted to be part of an operon (http://www.pseudomonas.com). Furthermore, HptB is a negative regulator of *rsmY* but not of *rsmZ* (Bordi et al., 2010). Similarly, the BfiSR two components system controls *rsmZ* but not *rsmY* via the RNase CafA (Petrova and Sauer, 2010). Remarkably, a mutation in *lptA*, encoding a lysophosphatidyl transferase, altered membrane fluidity (Baysse et al., 2005), led to increase expression of *rsmZ*, but not of *rsmY* (Baysse and O’Gara, 2007). Our study shows a c-di-GMP-dependent control of OprF, on *rsmZ* expression, but not on *rsmY* expression, providing a novel example of the dissociated control of the two sRNAs, *rsmY* and *rsmZ.*

How the c-di-GMP pool level is increased in the *oprF* mutant is not a trivial question. OprF is connecting the OM and the peptidoglycan layer and the absence of OprF provokes OM alterations (Rawling et al., 1998; Chevalier et al., 2000). It has been previously shown that H636 cells were shorter than the wild-type as judged by image analysis (Woodruff and Hancock, 1988) and by electron microscopy (Gotoh et al., 1989). Here we observe by SEM analyses that H636 cells were slightly thinner that H103 and H636O suggesting cell wall alterations. Cell morphology is, however, highly versatile and depends on growth conditions, as well as on the physiological state of the bacteria. However, despite suspected, it has never been clearly demonstrated that an *oprF* mutant encounters cell wall stress. Here, we show that the two ECFs sigma factors AlgU and SigX that are important for peptidoglycan and membrane homeostasis (Wood et al., 2006; Wood and Ohman, 2009; Boechat et al., 2013; Gicquel et al., 2013; Blanka et al., 2014), are active in the *oprF* mutant. Cell wall stress induced by D-cycloserin treatment (Wood and Ohman, 2009), or low shear modeled microgravity (Crabbé et al., 2010), is known to have similar effects on AlgU and SigX so one can hypothesize that deletion of *oprF* may result in a cell wall stress that then leads to production or activation of AlgU and SigX. Interestingly, two diguanylate cyclases, PA1181 and *adcA* were recently reported to belong to the SigX regulon of *P. aeruginosa* PA14 (Blanka et al., 2014). Accordingly, the two genes have lower expression levels in a *sigX*-deleted mutant of strain H103. While PA1181 and *adcA* expression is increased in the *oprF* mutant strain, the same was not true for PA2072, another c-di-GMP related protein belonging to the SigX regulon. Taken together, these data support the idea that *P. aeruginosa oprF* mutant displays higher levels of c-di-GMP as a consequence of the cell wall stress that the lack of OprF imposes to the cells. In addition to this, it is known that the two ECFs are involved in *P. aeruginosa* PAO1 biofilm formation (Bazire et al., 2010; Gicquel et al., 2013), so this study also highlights the possible role of cell envelope stress in biofilm formation. Our observation might in fact consolidate this concept, since an *ompA* mutant of *Escherichia coli* was shown to form sticky colonies by overproducing the exopolysaccharide cellulose through activation of the CpxRA TCS that responds to membrane stress (Ma et al., 2009). The bacteria might compensate the loss of the major OM protein OprF and the related membrane stress by producing an EPS that may stabilize the membrane, or protect itself from the environment.

This hypothesis is further strengthened by the fact that upon contact with surfaces, c-di-GMP is stimulated through the chemosensory-like surface-sensing system Wsp (Güvener and Harwood, 2007; O’Connor et al., 2012; Huangyutitham et al., 2013), though the exact nature of the signal remains obscure. Very recently, it was suggested that membrane alterations led to increase biofilm production through WspR activation resulting in c-di-GMP production (Blanka et al., 2015). In *Vibrio cholerae*, growth at low temperature modulates membrane fluidity and alters c-di-GMP signaling and biofilm formation (Townsley and Yildiz, 2015). Interestingly, Townsley and Yildiz (2015) show that temperature modulates c-di-GMP levels in a similar fashion in *P. aeruginosa* but not in the Gram-positive pathogen *Listeria monocytogenes*. Taken together, our study further supports the concept associated with these data and suggests that membrane alterations affect activity of enzymes involved in c-di-GMP metabolism, although the molecular mechanism remains to be elucidated.

The alternative or additional possibility that the effects caused by the absence of OprF result from something else other than OM disorganization cannot be disregarded, especially considering the role of OprF as a host immune system sensor (Wu et al., 2005; Mishra et al., 2015), more generally as an environmental sensor (Wagner et al., 2006; Fito-Boncompte et al., 2011). It is also conceivable that OprF-dependent c-di-GMP modulation is linked to its ability to transmit or to transduce unknown signals through the OM, which would then be detected by a sensor protein in the cytoplasmic membrane. Taken together, our data show for the first time that OprF is linked to c-di-GMP level modulations, through a direct (signaling) and/or indirect (envelope stress) mechanism.

## Author Contributions

Conceived and designed experiments: EB, JM, PL, AD, AF, SC.

Performed the experiments: EB, JM, RD, OM, LF-B, GG, AB, GB-W.

Analyzed the data: EB, JM, RD, PL, AD, AF, SC, JO, GB-W.

Contributed reagents/materials/analysis tools: OM, MB.

Wrote the paper: EB, JM, PL, AD, NO, MF, AF, GB-W, JO, SC.

All authors read and approved the final manuscript.

## Conflict of Interest Statement

The authors declare that the research was conducted in the absence of any commercial or financial relationships that could be construed as a potential conflict of interest.
